# 6-(4-Pyrid­yl)-3-(3,4,5-trimethoxy­phen­yl)-1,2,4-triazolo[3,4-*b*][1,3,4]thia­diazole

**DOI:** 10.1107/S1600536808023544

**Published:** 2008-07-31

**Authors:** Hai-Tang Du, Hai-Jun Du

**Affiliations:** a Institute of Natural Products, Research Center for Eco-Environmental Sciences, Guiyang College, Guiyang 550005, People’s Republic of China; b School of Chemistry and Environmental Sciences, Guizhou University for Nationalities, Guiyang 550025, People’s Republic of China

## Abstract

In the mol­ecule of the title compound, C_17_H_15_N_5_O_3_S, the planar central heterocylic ring system is oriented at dihedral angles of 5.32 (4) and 9.41 (4)°, respectively with respect to trimethoxy­phenyl and pyridine rings. Intra­molecular C—H⋯N, C—H⋯O and C—H⋯S hydrogen bonds result in the formation of a nearly planar six-membered ring, which is oriented at a dihedral angle of 3.07 (5)° with respect to the central heterocylic ring system, and non-planar six- and five-membered rings having twist and envelope conformations, respectively. In the crystal structure, inter­molecular C—H⋯N and C—H⋯O hydrogen bonds link the mol­ecules. There is a C—H⋯π contact between the pyridine ring and a methyl group and a π–π contact between the central heterocylic ring system and the trimethoxy­phenyl ring [centroid–centroid distance = 3.758 (1) Å].

## Related literature

For general background, see: Karabasanagouda *et al.* (2007 ▶); Mathew *et al.* (2007 ▶). For ring conformation puckering parameters, see: Cremer & Pople (1975 ▶). 
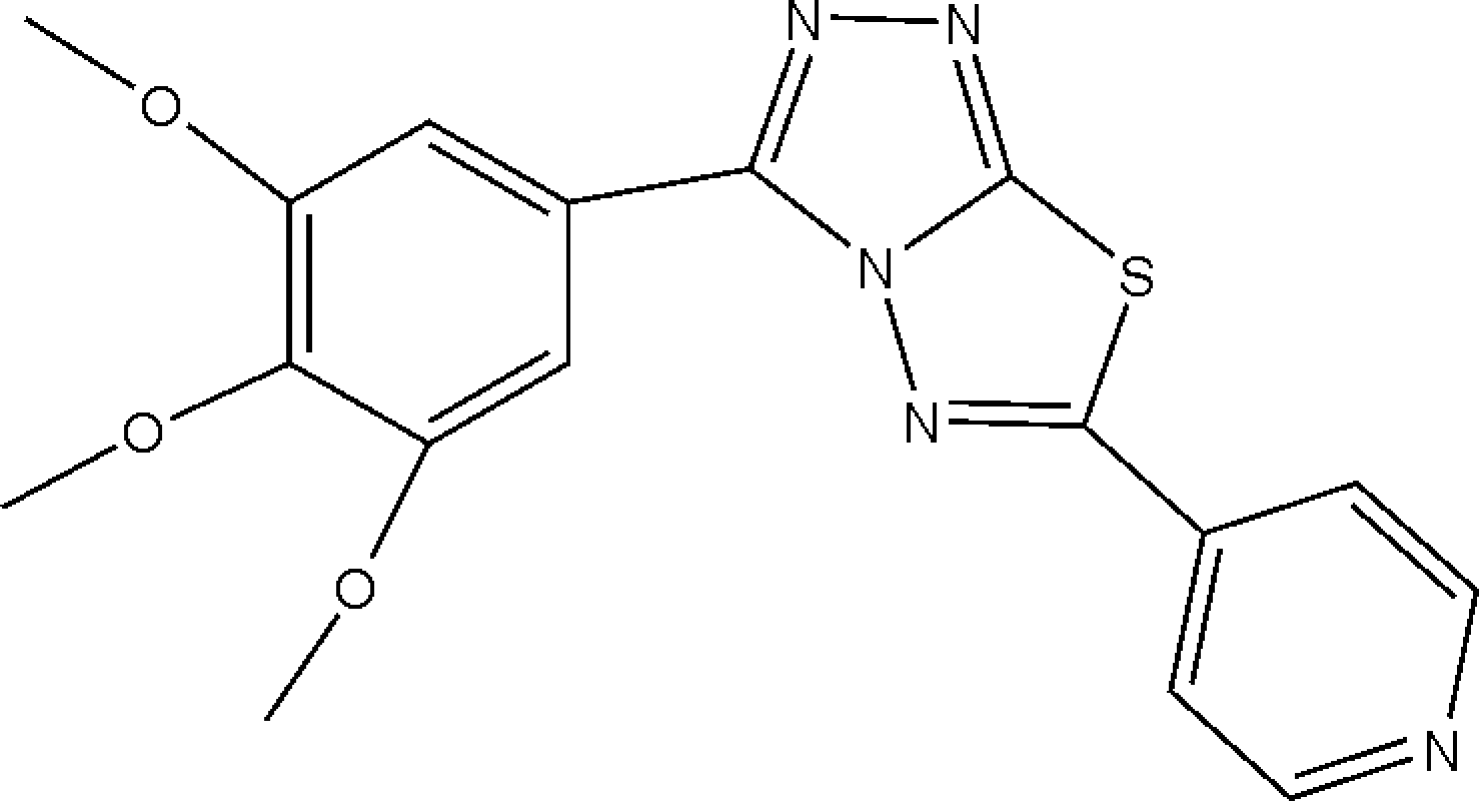


## Experimental

### 

#### Crystal data

C_17_H_15_N_5_O_3_S
*M* *_r_* = 369.40
Triclinic, 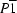

*a* = 7.7051 (15) Å
*b* = 8.6684 (17) Å
*c* = 13.851 (3) Å
α = 105.00 (3)°
β = 104.18 (3)°
γ = 96.58 (3)°
*V* = 850.6 (4) Å^3^
*Z* = 2
Mo *K*α radiation
μ = 0.22 mm^−1^
*T* = 113 (2) K
0.22 × 0.20 × 0.12 mm

#### Data collection

Rigaku Saturn CCD area-detector diffractometer
Absorption correction: multi-scan (*CrystalClear*; Rigaku/MSC, 2005 ▶) *T*_min_ = 0.953, *T*_max_ = 0.974
4917 measured reflections
2970 independent reflections
2467 reflections with *I* > 2σ(*I*)
*R*_int_ = 0.021

#### Refinement

*R*[*F* ^2^ > 2σ(*F* ^2^)] = 0.033
*wR*(*F* ^2^) = 0.100
*S* = 1.09
2970 reflections
238 parameters
H-atom parameters constrained
Δρ_max_ = 0.33 e Å^−3^
Δρ_min_ = −0.41 e Å^−3^

### 

Data collection: *CrystalClear* (Rigaku/MSC, 2005 ▶); cell refinement: *CrystalClear*; data reduction: *CrystalStructure* (Rigaku/MSC, 2005 ▶); program(s) used to solve structure: *SHELXS97* (Sheldrick, 2008 ▶); program(s) used to refine structure: *SHELXL97* (Sheldrick, 2008 ▶); molecular graphics: *SHELXTL* (Sheldrick, 2008 ▶); software used to prepare material for publication: *SHELXTL*.

## Supplementary Material

Crystal structure: contains datablocks I, global. DOI: 10.1107/S1600536808023544/hk2505sup1.cif

Structure factors: contains datablocks I. DOI: 10.1107/S1600536808023544/hk2505Isup2.hkl

Additional supplementary materials:  crystallographic information; 3D view; checkCIF report

## Figures and Tables

**Table 1:** Hydrogen-bond geometry (Å, °)

*D*—H⋯*A*	*D*—H	H⋯*A*	*D*⋯*A*	*D*—H⋯*A*
C2—H2⋯N4	0.93	2.32	3.016 (3)	131
C7—H7*B*⋯O1^i^	0.96	2.45	3.313 (3)	149
C8—H8*A*⋯O1	0.96	2.59	3.106 (3)	114
C8—H8*A*⋯N1^ii^	0.96	2.60	3.409 (3)	142
C14—H14⋯S1	0.93	2.80	3.185 (3)	106
C9—H9*A*⋯*Cg*3^iii^	0.96	3.07	3.974 (3)	157

Symmetry codes: (i) 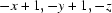; (ii) 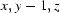; (iii) 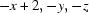. *Cg*3 is the centroid of the N5/C13–C17 pyridine ring.

**Fig. 1.**
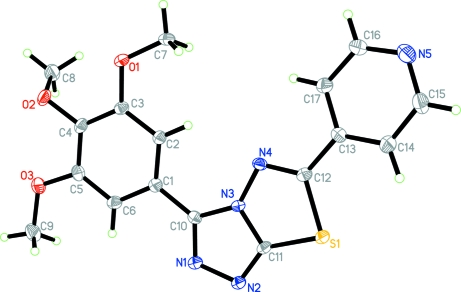
The molecular structure of the title molecule, with the atom-numbering scheme. Displacement ellipsoids are drawn at the 50% probability level.

**Fig. 2.**
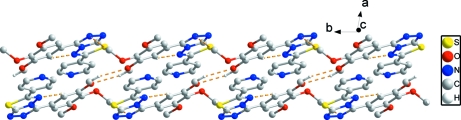
A partial packing diagram of the title compound. Hydrogen bonds are shown as dashed lines.

## supplementary crystallographic information

### Comment

1,2,4-Triazole and 1,3,4-thiadiazole represent one of the most biologically active classes of compounds, possessing a wide spectrum of activities. Various substituted 1,2,4-triazolo[3,4-b]-1,3,4-thiadiazoles are associated with diverse pharmacological activities such as antimicrobial (Karabasanagouda *et al.*,  2007) and anti-inflammatory activity (Mathew *et al.*,  2007). We report herein the crystal structure of the title compound.

In the molecule of the title compound (Fig. 1) the bond lengths and angles are within normal ranges. Rings A (C1-C6), B (N1-N3/C10/C11), C (S1/N3/N4/C11/C12) and D (N5/C13-C17) are, of course, planar, and the dihedral angles between them are A/B = 4.89 (6)°, A/C = 5.67 (5)°, A/D = 14.48 (5)°, B/C = 0.78 (5)°, B/D = 9.84 (5)° and C/D = 9.09 (4)°. So, rings B and C are nearly coplanar. The coplanar ring system is oriented with respect to rings A and D at dihedral angles of 5.32 (4)° and 9.41 (4)°. The intramolecular C-H···N, C-H···O and C-H···S hydrogen bonds (Table 1) result in the formation of nearly planar six-membered ring E (N3/N4/C1/C2/C10/H2) and non-planar six- and five-membered rings F (O1/O2/C3/C4/C8/H8A) and G (S1/C12-C14/H14). Ring E is oriented with respect to the planar central heterocylic ring system at a dihedral angle of 3.07 (5)°. Ring F has twisted [φ = -109.11 (2)°, θ = 117.46 (3)°] conformation, having total puckering amplitude, Q_T_, of 1.434 (3) Å (Cremer & Pople, 1975). Ring G adopts envelope conformation, with S1 atom displaced by 0.246 (3) Å from the plane of the other ring atoms.

In the crystal structure, intermolecular C-H···N and C-H···O hydrogen bonds link the molecules (Fig. 2), in which they may be effective in the stabilization of the structure. A C—H···π contact (Table 1) between the pyridine ring and the methyl group and a π—π contact between C and A rings Cg1···Cg4^i^ [symmetry code: (i) 2 - x, -y, -z, where Cg1 and Cg4 are centroids of the rings C (S1/N3/N4/C11/C12) and A (C1-C6), respectively] further stabilize the structure, with centroid-centroid distance of 3.758 (1) Å.

### Experimental

For the preparation of the title compound, 4-amino-5-(3,4,5-trimethoxyphenyl) -4H-1,2,4-triazole-3-thiol (0.01 M) and isonicotinic acid (0.01 M) were dissolved in dry phosphorous oxychloride (10 ml). The resulted solution was further heated under reflux for 7 h. The reaction mixture was cooled to room temperature and the mixture was gradually poured onto crushed ice with stirring. Finally, powdered potassium carbonate and the required amount of solid potassium hydroxide were added until the pH of the mixture was raised to 8, to remove the excess of phosphorous oxychloride. The mixture was allowed to stand overnight and the solid was separated. It was filtered, washed with cold water, and then dried. Crystals suitable for X-ray analysis were obtained by the recrystallization of the solid residue from a mixture of N,N-dimethylformamide/ethanol (1:1) by slow evaporation at room temperature.

### Refinement

H atoms were positioned geometrically, with C-H = 0.93 and 0.96 Å for aromatic and methyl H, respectively, and constrained to ride on their parent atoms with U_iso_(H) = xU_eq_(C), where x = 1.5 for methyl H and x = 1.2 for aromatic H atoms.

### Crystal data

**Table:**

C_17_H_15_N_5_O_3_S	*Z* = 2
*M**_r_* = 369.40	*F*_000_ = 384
Triclinic, *P*1	*D*_x_ = 1.442 Mg m^−^^3^
Hall symbol: -P 1	Melting point: 447 K
*a* = 7.7051 (15) Å	Mo *K*α radiation λ = 0.71073 Å
*b* = 8.6684 (17) Å	Cell parameters from 2198 reflections
*c* = 13.851 (3) Å	θ = 2.5–27.5º
α = 105.00 (3)º	µ = 0.22 mm^−^^1^
β = 104.18 (3)º	*T* = 113 (2) K
γ = 96.58 (3)º	Prism, colorless
*V* = 850.6 (4) Å^3^	0.22 × 0.20 × 0.12 mm

### Data collection

**Table:**

Rigaku Saturn CCD area-detector diffractometer	2970 independent reflections
Radiation source: rotating anode	2467 reflections with *I* > 2σ(*I*)
Monochromator: confocal	*R*_int_ = 0.021
*T* = 113(2) K	θ_max_ = 25.0º
ω scans	θ_min_ = 1.6º
Absorption correction: multi-scan(CrystalClear; Rigaku/MSC, 2005)	*h* = −9→8
*T*_min_ = 0.953, *T*_max_ = 0.974	*k* = −5→10
4917 measured reflections	*l* = −16→16

### Refinement

**Table:**

Refinement on *F*^2^	Secondary atom site location: difference Fourier map
Least-squares matrix: full	Hydrogen site location: inferred from neighbouring sites
*R*[*F*^2^ > 2σ(*F*^2^)] = 0.033	H-atom parameters constrained
*wR*(*F*^2^) = 0.100	*w* = 1/[σ^2^(*F*_o_^2^) + (0.0666*P*)^2^] where *P* = (*F*_o_^2^ + 2*F*_c_^2^)/3
*S* = 1.09	(Δ/σ)_max_ = 0.001
2970 reflections	Δρ_max_ = 0.33 e Å^−^^3^
238 parameters	Δρ_min_ = −0.41 e Å^−^^3^
Primary atom site location: structure-invariant direct methods	Extinction correction: none

### Special details

**Table:**

Geometry. All e.s.d.'s (except the e.s.d. in the dihedral angle between two l.s. planes) are estimated using the full covariance matrix. The cell e.s.d.'s are taken into account individually in the estimation of e.s.d.'s in distances, angles and torsion angles; correlations between e.s.d.'s in cell parameters are only used when they are defined by crystal symmetry. An approximate (isotropic) treatment of cell e.s.d.'s is used for estimating e.s.d.'s involving l.s. planes.
Refinement. Refinement of F^2^ against ALL reflections. The weighted R-factor wR and goodness of fit S are based on F^2^, conventional R-factors R are based on F, with F set to zero for negative F^2^. The threshold expression of F^2^ > 2sigma(F^2^) is used only for calculating R-factors(gt) etc. and is not relevant to the choice of reflections for refinement. R-factors based on F^2^ are statistically about twice as large as those based on F, and R- factors based on ALL data will be even larger.

### Fractional atomic coordinates and isotropic or equivalent isotropic displacement parameters (Å^2^)

**Table:**

	*x*	*y*	*z*	*U*_iso_*/*U*_eq_	
S1	0.12462 (5)	1.42520 (5)	0.15970 (3)	0.01718 (15)	
O1	0.42347 (15)	0.65186 (14)	−0.05689 (9)	0.0207 (3)	
O2	0.38351 (15)	0.55944 (14)	−0.26365 (10)	0.0221 (3)	
O3	0.22791 (16)	0.72787 (15)	−0.38536 (9)	0.0237 (3)	
N1	0.06954 (17)	1.22162 (16)	−0.13378 (11)	0.0173 (3)	
N2	0.04356 (18)	1.35552 (17)	−0.06135 (11)	0.0174 (3)	
N3	0.16107 (16)	1.18786 (16)	0.01937 (10)	0.0137 (3)	
N4	0.22454 (17)	1.14340 (16)	0.10761 (11)	0.0153 (3)	
N5	0.3721 (2)	1.2020 (2)	0.49150 (12)	0.0271 (4)	
C1	0.2006 (2)	0.9733 (2)	−0.13125 (13)	0.0154 (4)	
C2	0.2800 (2)	0.8834 (2)	−0.06844 (13)	0.0153 (4)	
H2	0.2920	0.9157	0.0027	0.018*	
C3	0.3410 (2)	0.7454 (2)	−0.11299 (13)	0.0159 (4)	
C4	0.3211 (2)	0.6951 (2)	−0.21931 (14)	0.0172 (4)	
C5	0.2417 (2)	0.7869 (2)	−0.28176 (13)	0.0183 (4)	
C6	0.1823 (2)	0.9264 (2)	−0.23794 (13)	0.0176 (4)	
H6	0.1308	0.9879	−0.2793	0.021*	
C7	0.4203 (2)	0.6867 (2)	0.04955 (13)	0.0197 (4)	
H7A	0.4866	0.7948	0.0879	0.030*	
H7B	0.4757	0.6100	0.0798	0.030*	
H7C	0.2963	0.6786	0.0522	0.030*	
C8	0.2630 (3)	0.4101 (2)	−0.27988 (15)	0.0273 (4)	
H8A	0.2430	0.4057	−0.2148	0.041*	
H8B	0.3168	0.3198	−0.3067	0.041*	
H8C	0.1488	0.4049	−0.3290	0.041*	
C9	0.1605 (3)	0.8243 (2)	−0.45012 (15)	0.0285 (4)	
H9A	0.0370	0.8318	−0.4506	0.043*	
H9B	0.1644	0.7747	−0.5199	0.043*	
H9C	0.2347	0.9313	−0.4235	0.043*	
C10	0.1416 (2)	1.1222 (2)	−0.08494 (13)	0.0145 (3)	
C11	0.1006 (2)	1.33163 (19)	0.03000 (13)	0.0151 (4)	
C12	0.2130 (2)	1.2572 (2)	0.18640 (13)	0.0156 (4)	
C13	0.2677 (2)	1.2409 (2)	0.29190 (13)	0.0163 (4)	
C14	0.2786 (2)	1.3663 (2)	0.37991 (14)	0.0225 (4)	
H14	0.2510	1.4657	0.3738	0.027*	
C15	0.3307 (2)	1.3421 (2)	0.47678 (15)	0.0265 (4)	
H15	0.3376	1.4276	0.5351	0.032*	
C16	0.3604 (2)	1.0826 (2)	0.40556 (14)	0.0242 (4)	
H16	0.3891	0.9846	0.4138	0.029*	
C17	0.3089 (2)	1.0943 (2)	0.30574 (14)	0.0212 (4)	
H17	0.3017	1.0063	0.2488	0.025*	

### Atomic displacement parameters (Å^2^)

**Table:**

	*U*^11^	*U*^22^	*U*^33^	*U*^12^	*U*^13^	*U*^23^
S1	0.0206 (2)	0.0148 (2)	0.0170 (2)	0.00664 (17)	0.00523 (17)	0.00510 (18)
O1	0.0273 (6)	0.0190 (7)	0.0201 (7)	0.0131 (5)	0.0084 (5)	0.0081 (5)
O2	0.0265 (6)	0.0152 (6)	0.0284 (7)	0.0078 (5)	0.0154 (5)	0.0040 (6)
O3	0.0328 (7)	0.0239 (7)	0.0151 (6)	0.0079 (5)	0.0091 (5)	0.0039 (6)
N1	0.0198 (7)	0.0153 (7)	0.0182 (8)	0.0056 (6)	0.0056 (6)	0.0064 (6)
N2	0.0213 (7)	0.0153 (7)	0.0172 (8)	0.0069 (6)	0.0057 (6)	0.0057 (6)
N3	0.0149 (7)	0.0117 (7)	0.0155 (7)	0.0040 (5)	0.0037 (5)	0.0055 (6)
N4	0.0161 (7)	0.0168 (7)	0.0148 (7)	0.0047 (6)	0.0039 (5)	0.0077 (6)
N5	0.0300 (8)	0.0316 (9)	0.0211 (9)	0.0062 (7)	0.0071 (7)	0.0105 (8)
C1	0.0126 (8)	0.0155 (9)	0.0177 (9)	0.0019 (6)	0.0043 (6)	0.0048 (7)
C2	0.0151 (8)	0.0146 (8)	0.0161 (9)	0.0027 (6)	0.0048 (6)	0.0039 (7)
C3	0.0127 (8)	0.0150 (9)	0.0206 (9)	0.0033 (6)	0.0045 (6)	0.0064 (7)
C4	0.0164 (8)	0.0128 (8)	0.0229 (9)	0.0025 (7)	0.0089 (7)	0.0030 (7)
C5	0.0169 (8)	0.0203 (9)	0.0169 (9)	0.0006 (7)	0.0061 (7)	0.0040 (8)
C6	0.0164 (8)	0.0181 (9)	0.0193 (9)	0.0048 (7)	0.0041 (7)	0.0074 (8)
C7	0.0232 (9)	0.0184 (9)	0.0204 (9)	0.0080 (7)	0.0050 (7)	0.0097 (8)
C8	0.0395 (11)	0.0180 (9)	0.0270 (10)	0.0051 (8)	0.0160 (8)	0.0050 (8)
C9	0.0341 (10)	0.0347 (11)	0.0181 (10)	0.0075 (9)	0.0088 (8)	0.0088 (9)
C10	0.0130 (7)	0.0151 (8)	0.0150 (8)	0.0003 (6)	0.0034 (6)	0.0052 (7)
C11	0.0131 (8)	0.0122 (8)	0.0212 (9)	0.0042 (6)	0.0059 (7)	0.0055 (7)
C12	0.0127 (8)	0.0147 (8)	0.0197 (9)	0.0027 (6)	0.0040 (6)	0.0057 (7)
C13	0.0124 (8)	0.0203 (9)	0.0166 (9)	0.0026 (7)	0.0036 (6)	0.0069 (7)
C14	0.0272 (9)	0.0187 (9)	0.0218 (10)	0.0048 (8)	0.0083 (7)	0.0050 (8)
C15	0.0314 (10)	0.0268 (11)	0.0191 (10)	0.0016 (8)	0.0086 (8)	0.0035 (8)
C16	0.0273 (9)	0.0259 (10)	0.0227 (10)	0.0085 (8)	0.0070 (7)	0.0114 (8)
C17	0.0224 (9)	0.0214 (9)	0.0203 (9)	0.0076 (7)	0.0047 (7)	0.0070 (8)

### Geometric parameters (Å, °)

**Table:**

S1—C11	1.7226 (18)	C3—C4	1.387 (2)
S1—C12	1.7615 (17)	C4—C5	1.402 (2)
O1—C3	1.3664 (19)	C5—C6	1.387 (2)
O1—C7	1.433 (2)	C6—H6	0.9300
O2—C4	1.376 (2)	C7—H7A	0.9600
O2—C8	1.435 (2)	C7—H7B	0.9600
O3—C5	1.366 (2)	C7—H7C	0.9600
O3—C9	1.426 (2)	C8—H8A	0.9600
N1—C10	1.317 (2)	C8—H8B	0.9600
N1—N2	1.394 (2)	C8—H8C	0.9600
N2—C11	1.312 (2)	C9—H9A	0.9600
N3—C11	1.365 (2)	C9—H9B	0.9600
N3—N4	1.3694 (18)	C9—H9C	0.9600
N3—C10	1.372 (2)	C12—C13	1.467 (2)
N4—C12	1.297 (2)	C13—C14	1.385 (2)
N5—C16	1.336 (2)	C13—C17	1.393 (2)
N5—C15	1.343 (2)	C14—C15	1.381 (2)
C1—C2	1.392 (2)	C14—H14	0.9300
C1—C6	1.394 (2)	C15—H15	0.9300
C1—C10	1.460 (2)	C16—C17	1.376 (2)
C2—C3	1.387 (2)	C16—H16	0.9300
C2—H2	0.9300	C17—H17	0.9300
			
C11—S1—C12	87.35 (8)	O2—C8—H8B	109.5
C3—O1—C7	116.86 (13)	H8A—C8—H8B	109.5
C4—O2—C8	113.05 (12)	O2—C8—H8C	109.5
C5—O3—C9	116.86 (14)	H8A—C8—H8C	109.5
C10—N1—N2	109.38 (13)	H8B—C8—H8C	109.5
C11—N2—N1	106.05 (13)	O3—C9—H9A	109.5
C11—N3—N4	117.95 (14)	O3—C9—H9B	109.5
C11—N3—C10	106.53 (13)	H9A—C9—H9B	109.5
N4—N3—C10	135.51 (13)	O3—C9—H9C	109.5
C12—N4—N3	107.80 (13)	H9A—C9—H9C	109.5
C16—N5—C15	116.09 (16)	H9B—C9—H9C	109.5
C2—C1—C6	120.81 (15)	N1—C10—N3	107.71 (14)
C2—C1—C10	120.03 (15)	N1—C10—C1	126.81 (15)
C6—C1—C10	119.11 (15)	N3—C10—C1	125.40 (14)
C3—C2—C1	119.36 (16)	N2—C11—N3	110.32 (15)
C3—C2—H2	120.3	N2—C11—S1	139.95 (13)
C1—C2—H2	120.3	N3—C11—S1	109.73 (12)
O1—C3—C2	123.22 (15)	N4—C12—C13	120.08 (15)
O1—C3—C4	116.10 (14)	N4—C12—S1	117.15 (12)
C2—C3—C4	120.68 (15)	C13—C12—S1	122.75 (13)
O2—C4—C3	120.89 (15)	C14—C13—C17	117.81 (16)
O2—C4—C5	119.60 (15)	C14—C13—C12	122.40 (16)
C3—C4—C5	119.48 (15)	C17—C13—C12	119.78 (16)
O3—C5—C6	124.38 (15)	C15—C14—C13	119.24 (17)
O3—C5—C4	115.23 (15)	C15—C14—H14	120.4
C6—C5—C4	120.39 (15)	C13—C14—H14	120.4
C5—C6—C1	119.26 (15)	N5—C15—C14	123.67 (18)
C5—C6—H6	120.4	N5—C15—H15	118.2
C1—C6—H6	120.4	C14—C15—H15	118.2
O1—C7—H7A	109.5	N5—C16—C17	124.66 (17)
O1—C7—H7B	109.5	N5—C16—H16	117.7
H7A—C7—H7B	109.5	C17—C16—H16	117.7
O1—C7—H7C	109.5	C16—C17—C13	118.51 (18)
H7A—C7—H7C	109.5	C16—C17—H17	120.7
H7B—C7—H7C	109.5	C13—C17—H17	120.7
O2—C8—H8A	109.5		
			
C10—N1—N2—C11	−0.40 (17)	N4—N3—C10—C1	−3.7 (3)
C11—N3—N4—C12	−0.60 (19)	C2—C1—C10—N1	177.96 (15)
C10—N3—N4—C12	178.75 (15)	C6—C1—C10—N1	0.3 (2)
C6—C1—C2—C3	−0.1 (2)	C2—C1—C10—N3	1.7 (2)
C10—C1—C2—C3	−177.80 (14)	C6—C1—C10—N3	−176.04 (13)
C7—O1—C3—C2	9.6 (2)	N1—N2—C11—N3	−0.34 (17)
C7—O1—C3—C4	−170.70 (13)	N1—N2—C11—S1	179.14 (15)
C1—C2—C3—O1	178.75 (13)	N4—N3—C11—N2	−179.55 (12)
C1—C2—C3—C4	−1.0 (2)	C10—N3—C11—N2	0.92 (17)
C8—O2—C4—C3	79.22 (19)	N4—N3—C11—S1	0.80 (17)
C8—O2—C4—C5	−102.87 (18)	C10—N3—C11—S1	−178.73 (9)
O1—C3—C4—O2	−0.6 (2)	C12—S1—C11—N2	179.97 (19)
C2—C3—C4—O2	179.15 (14)	C12—S1—C11—N3	−0.55 (11)
O1—C3—C4—C5	−178.51 (14)	N3—N4—C12—C13	178.53 (13)
C2—C3—C4—C5	1.2 (2)	N3—N4—C12—S1	0.12 (16)
C9—O3—C5—C6	5.0 (2)	C11—S1—C12—N4	0.26 (13)
C9—O3—C5—C4	−175.42 (14)	C11—S1—C12—C13	−178.10 (13)
O2—C4—C5—O3	2.1 (2)	N4—C12—C13—C14	172.23 (15)
C3—C4—C5—O3	180.00 (13)	S1—C12—C13—C14	−9.5 (2)
O2—C4—C5—C6	−178.33 (14)	N4—C12—C13—C17	−8.5 (2)
C3—C4—C5—C6	−0.4 (2)	S1—C12—C13—C17	169.79 (12)
O3—C5—C6—C1	178.88 (14)	C17—C13—C14—C15	0.6 (2)
C4—C5—C6—C1	−0.7 (2)	C12—C13—C14—C15	179.86 (14)
C2—C1—C6—C5	1.0 (2)	C16—N5—C15—C14	0.0 (3)
C10—C1—C6—C5	178.66 (14)	C13—C14—C15—N5	−0.1 (3)
N2—N1—C10—N3	0.96 (17)	C15—N5—C16—C17	−0.4 (3)
N2—N1—C10—C1	−175.87 (14)	N5—C16—C17—C13	0.8 (3)
C11—N3—C10—N1	−1.14 (17)	C14—C13—C17—C16	−0.9 (2)
N4—N3—C10—N1	179.45 (15)	C12—C13—C17—C16	179.80 (14)
C11—N3—C10—C1	175.75 (14)		

### Hydrogen-bond geometry (Å, °)

**Table:**

*D*—H···*A*	*D*—H	H···*A*	*D*···*A*	*D*—H···*A*
C2—H2···N4	0.93	2.32	3.016 (3)	131
C7—H7B···O1^i^	0.96	2.45	3.313 (3)	149
C8—H8A···O1	0.96	2.59	3.106 (3)	114
C8—H8A···N1^ii^	0.96	2.60	3.409 (3)	142
C14—H14···S1	0.93	2.80	3.185 (3)	106
C9—H9A···Cg3^iii^	0.96	3.07	3.974 (3)	157

Symmetry codes: (i) −*x*+1, −*y*+1, −*z*; (ii) *x*, *y*−1, *z*; (iii) −*x*+2, −*y*, −*z*.
